# Arteriovenous malformation of the thyroid gland as a very rare cause of mechanical neck syndrome: a case report

**DOI:** 10.1186/1752-1947-9-3

**Published:** 2015-02-02

**Authors:** Monika Černá, Vladislav Třeška, Michal Krčma, Ondřej Daum, František Šlauf

**Affiliations:** 1 Clinic of Surgery, Plzeň Teaching Hospital and Charles University Medical Faculty, Alej Svobody 80, 304 60 Plzeň – Lochotín, Czech Republic; 2 1st Clinic of Internal Medicine, Plzeň Teaching Hospital and Charles University Medical Faculty, Alej Svobody 80, 304 60 Plzeň – Lochotín, Czech Republic; 3 Šikl Institute of Pathology and Anatomy, Plzeň Teaching Hospital and Charles University Medical Faculty, Edvarda Beneše 1128/13, 305 99 Plzeň-Bory, Czech Republic; 4 Clinic of Radiology, Plzeň Teaching Hospital and Charles University Medical Faculty, Alej Svobody 80, 304 60 Plzeň-Lochotín, Czech Republic

**Keywords:** Arteriovenous malformation, Intraarterial embolisation, Mechanical neck syndrome, Thyroid gland

## Abstract

**Introduction:**

Vascular malformations of the thyroid gland represent a very rare, often accidentally diagnosed, disease that in the case of eufunctional goitre may be the cause of mechanical neck syndrome. The authors present here the complex differential-diagnosis and treatment approach and stress the importance of histopathology for determining the final diagnosis.

**Case presentation:**

Using various imaging methods (ultrasound, multidetector computed tomography of the neck), the cause of breathing difficulties in a 64-year-old old man from the Czech Republic with normal thyroid gland function was found to be an arteriovenous malformation of the left lobe of his thyroid gland, 80×70×55mm in size, reaching retrosternally between the major arteries branching from his aortic arch and displacing his trachea 10mm to the right. In preparation for surgery, he underwent a radio-interventional procedure with embolisation of the arteries supplying the left lobe. This was followed by a lobectomy on the left via a partial sternotomy. The definitive histology result confirmed that the arteriovenous malformation was the benign cause of the mechanical neck syndrome.

**Conclusions:**

The case report presented here extends the differential diagnostic options in cases of mechanical neck syndrome. It describes a very rare disease of the thyroid gland, which prior to surgery may arouse suspicion of malignancy. It stresses the importance of close team cooperation between the endocrinologist, interventional radiologist and surgeon within the framework of preoperative diagnosis as well as preparation for surgery. Determination of the definitive histopathological diagnosis requires a pathologist experienced in such issues.

## Introduction

The thyroid gland (TG) belongs to a group of endocrine organs, which produce hormones that participate in the management of many metabolic processes. Therefore, most diseases affecting this gland frequently involve functional disorders in the sense of either hypo or hyperfunction (hypothyroidism and hyperthyroidism respectively), and their treatment falls within the domain of endocrinologists. Such patients become candidates for surgical treatment only when conservative (medicamentous) treatment fails or when they develop mechanical neck syndrome, which may occur even in the case of glands with normal secretory activity (eufunctional goitre). A second large group of TG diseases involves malignant tumours, which from the aspect of radicality always require surgical treatment. A less frequent group of TG diseases involves inflammation and these diseases are again managed by endocrinologists and may be the cause of secondary functional disorders.

This article presents a very rare case report of a 64-year-old man with an unusual underlying cause of mechanical neck syndrome, demonstrating the complex differential-diagnostic and therapeutic approach along with the histopathological determination of the final diagnosis.

## Case presentation

**Figure 1:**
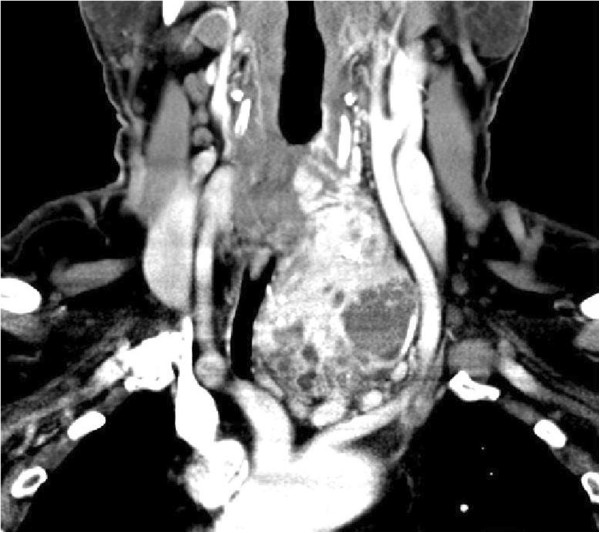
**Computed tomography of the neck-coronal.** Bulky left thyroid gland lobe displacing the branching arteries of the aortic arch and deviating the trachea to the right.

A 64-year-old man from the Czech Republic with a history of hypertension was first examined by a cardiologist for intermittent cough, mild dyspnoea on exertion and non-constant hoarseness. He underwent an X-ray examination, which inadvertently revealed deviation of his trachea 10mm to the right. An examination specifically targeting his TG revealed the presence of goitre, with significant predominance of the left lobe. He was referred for specialist examination by an endocrinologist who performed an ultrasound (USG) of his neck. The right lobe of his TG was 20×21×49mm in size with normal findings and the left lobe of 80×70×55mm was described as hypoechogenic, lobulated, hypervascularised with propagation retrosternally. It included several cystic lesions and a vascular plexus located in its lower section with the diameter of its arteries reaching up to 12mm. No cervical lymphadenopathy was present. In order to further clarify these findings, the patient underwent multidetector computed tomography (MDCT) of his neck focusing on the TG. This described the left lobe as reaching retrosternally, significantly deforming and compressing the trachea with a large nodule located at its inferior pole, displacing the arteries of the aortic arch, with which it was in close contact (Figure 1). The MDCT findings did not rule out the possibility of an arteriovenous malformation (AVM) originating from the principal arterial trunks of the aortic arch and left subclavian or brachiocephalic veins. The presence of multiple venous plexuses up to 20mm in width was described at the lower edge of the nodule in the upper mediastinum. According to the computed tomography (CT), the right lobe demonstrated no pathological findings; cervical lymphadenopathy was present but the lymph nodes were of benign appearance. The laboratory values of TG hormones were within physiological range.

**Figure 2:**
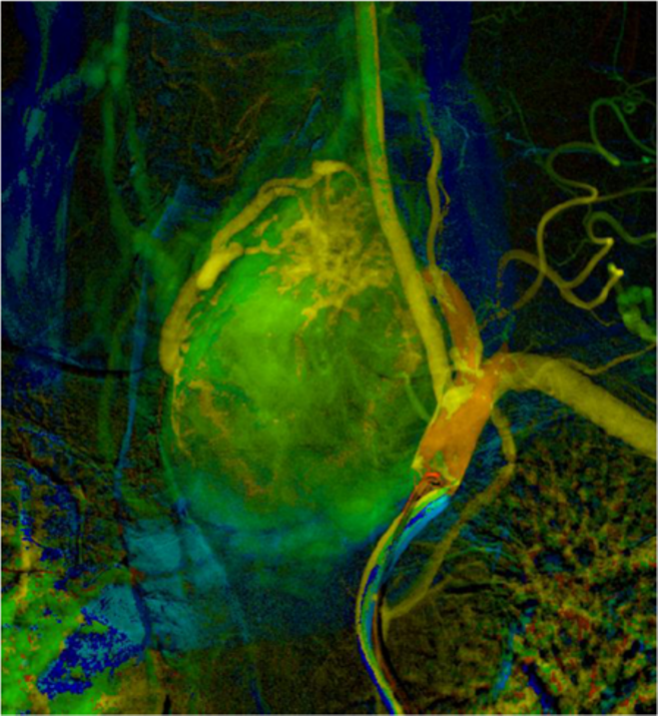
**Digital subtraction angiography imaging of the arteriovenous malformation vascular supply from the truncus thyrocervicalis and superior thyroid artery on the left.**

**Figure 3:**
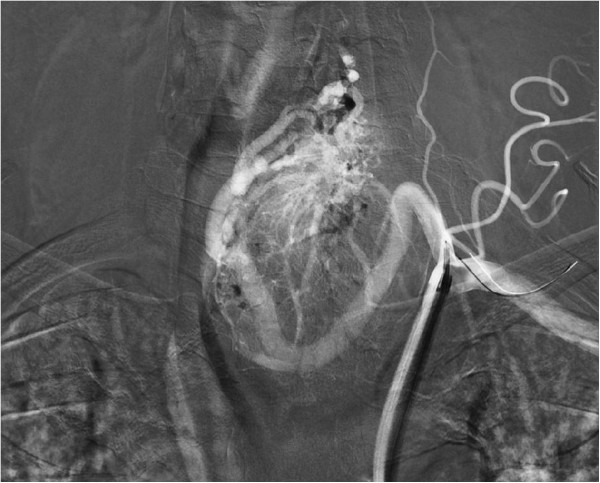
**Angiography.** Selective injection of contrast dye into arteriovenous malformation prior to embolisation.

**Figure 4:**
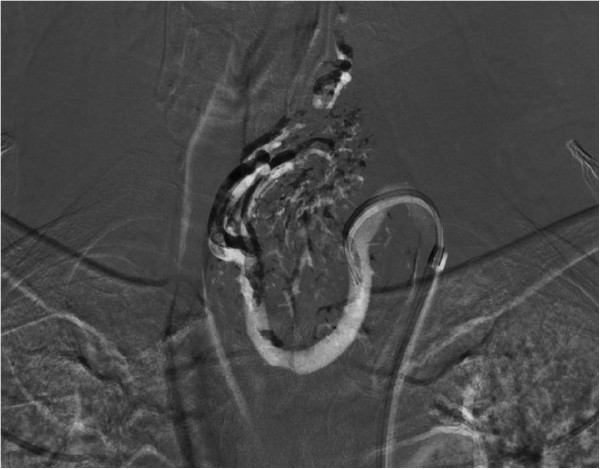
**The resulting effect of Histoacryl® radio-interventional application.**

**Figure 5:**
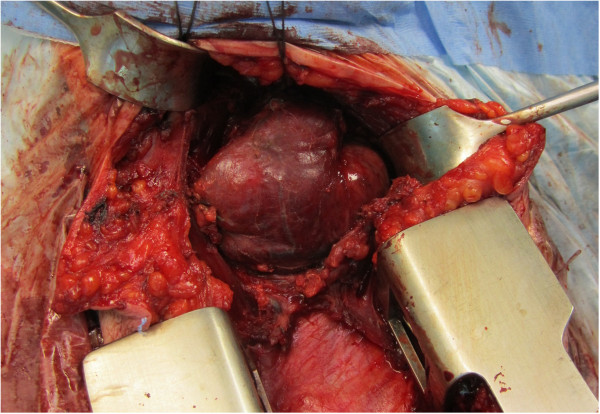
**Perioperative findings of an arteriovenous malformation of the thyroid gland left lobe.** Approach using the collar incision and partial sternotomy.

The endocrinologist referred the patient to the Clinic of Surgery for surgical treatment. The clinical presentation predominantly involved an enlarged TG left lobe with the presence of an audible murmur in this region on auscultation. In view of the suspected AVM and thus the high risk of perioperative serious, poorly manageable bleeding in the area of his upper mediastinum, the patient underwent a radio-interventional procedure via his right common femoral artery, which uncovered the presence of two massive arteries supplying the lesion, branching from the truncus thyrocervicalis and superior thyroid artery on the left (Figure 2). Both arteries supplying the lesion were embolised selectively using Histoacryl® (B Braun Melsungen AG, Melsungen, Germany; Figures 3 and 4). The procedure was totally uncomplicated. Four days later, he underwent surgery; lobectomy of his TG left lobe was performed via a partial sternotomy (Figure 5). In concurrence with the imaging methods, the nodule located in the lower pole of the left lobe extended to his aortic arch. It was surrounded by multiple vascular plexuses, which were ligated during the lobectomy. Following the prior embolisation, several of the vessels were already thrombosed, which significantly reduced any perioperative bleeding and technically facilitated orientation within the operating field. Nonetheless, the nodule itself as well as the whole TG lobe was very tough and fibrous and both adhered firmly to his trachea and short neck muscles. In the given terrain, it was impossible to identify the course of the recurrent laryngeal nerve and thus surgery proceeded closely along the TG capsule. Based on the agreement signed by the patient before the operation, lobectomy of the left lobe was performed and the right lobe was preserved. The latter was of normal appearance and consistence. If a malignancy had been described in the definitive histopathological findings, we would have subsequently performed lobectomy on the right. The perioperative blood loss was up to 200mL. There were no complications during the postoperative course. Phonation and respiration were normal from the very beginning. The laboratory values of calcium and phosphate were satisfactory. The blood loss was minimal and he did not require any transfusions. He was discharged home on the 7th postoperative day. He came in for his first check-up after 10 days and was found to be completely well, without any complaints. The wound had healed by primary intention and he was referred for further follow-up back to the treating endocrinologist. One month after surgery, substitution maintenance therapy was started to prevent compensatory hypertrophy of the contralateral lobe.

**Figure 6:**
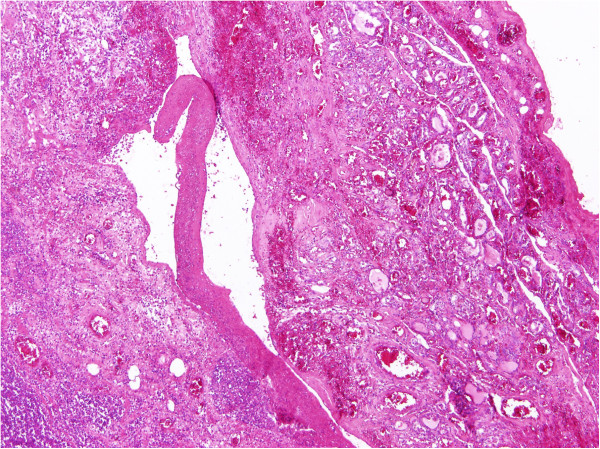
**Histopathological picture (hematoxylin and eosin, 40×).** On the right side, the residual atrophic thyroid tissue can be seen. In the centre, there is an adjacent malformed vessel containing a blood clot within its lumen. The left side of the picture consists of the nodule with regressive changes to its parenchyma.

**Figure 7:**
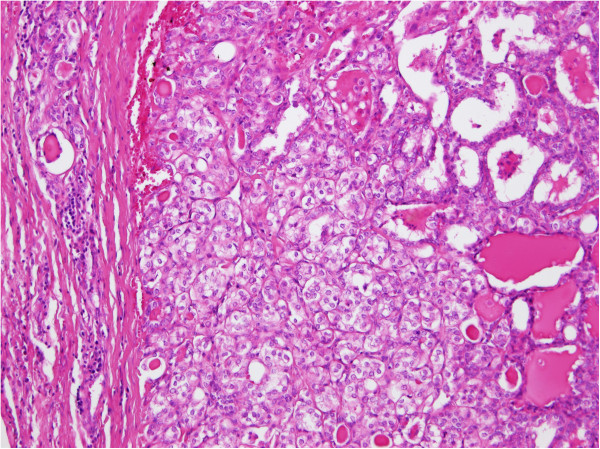
**Histopathological finding in detail (hematoxylin and eosin, 200×).** The intimate relationship between the clear cell (centre) and papillary (right) components of the nodule.

**Figure 8:**
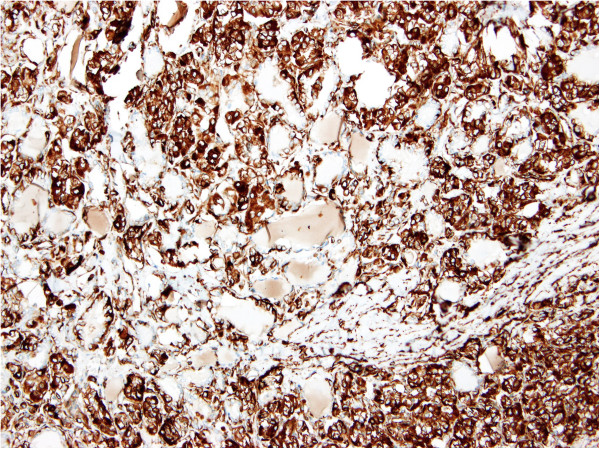
**Histopathological examination (vimentin, 200×).** Diffuse positivity of vimentin expression in the nodule-forming cells.

The histological examination showed a highly unusual lesion consisting of an AVM with obturation of certain arteries following embolisation: partially necrotised TG tissue with dystrophic calcifications and partially viable parenchyma with microfollicles from thyrocytes (Figures 6, 7 and 8). Despite the great similarity with certain tumours (especially papillary carcinoma) or metastasis of clear cell renal carcinoma, the pathologist concluded that this was a case of benign nodular hyperplasia of the TG parenchyma within the terrain of AVM.

## Discussion

AVM refers to congenital convolution of arteries and veins, which communicate directly and have no embedded capillary bed. This develops due to a disorder of differentiation during the development of the vascular bed. Because resistance of the capillary system is lacking in AVM, blood flow through the malformation is very high; the supplying arteries are usually greatly distended and coiled. The AVM itself may possess several arterial sources originating from a magistral vascular bed. The veins draining the AVM are wide; their walls are altered by the high blood flow rate: a state called arterialisation. An AVM may occur in practically any part of the body and in all organ systems. Depending on its location, it may be completely asymptomatic or it may present as bleeding, pain or symptoms arising from compression of the surrounding structures.

Asymptomatic AVMs are usually diagnosed by accident during examination of organ systems and depending on their size they are subject to further follow-up. In symptomatic patients with negative physical findings, they are primarily uncovered by USG. Verification of suspected AVM (and potential subsequent embolisation) is performed with the aid of digital subtraction angiography (DSA), an invasive method with the option of selective contrast dye application and imaging of arteries supplying the lesion [1]. Today, computed tomography angiography (CTAG) with intravenous administration of contrast dye provides a precise image of the vascular supply and its relationship with magistral arteries. DSA retains its place rather as a diagnostic-therapeutic method [2]. Depending on the location and need to display the relationship between the vascular malformation and soft tissue structures, magnetic resonance imaging (MRI) also offers clear information to this end [3]. We did not use this method in our case, given the clear MDCT findings. Decisions regarding treatment are made on the basis of the graphic image.

Therapeutic options include radio-interventional, surgical and combined approaches depending on the character and location of the AVM [4]. Basically, combined treatment is preferred. This refers to the reduction of blood flow through the AVM using radio-interventional selective embolisation of supply arteries with the aid of tissue adhesives or titanium spirals with subsequent surgical removal of the affected TG lobe, as was done in our case. Successful embolisation significantly reduces blood flow and facilitates surgical intervention particularly in poorly accessible locations and, in general, it reduces the risk of surgery as well as postoperative complications. It is recommended to perform surgery within 3 days of embolisation as neovascularisation of the AVM occurs 10 days after this.

AVMs of the TG are, in general, rare diseases and their preoperative diagnosis is usually difficult. Only one work has been published worldwide to date, by the Spanish authors Lizarralde and Diaz-Cano describing the issue discussed here involving two patients [5]. Small AVMs may be clinically asymptomatic and detected only when the TG is examined because of its suspected disorder or preventively, for example in the case of a positive family history of TG disease. Larger AVMs most frequently present as a mechanical syndrome; first, dysphagia and later on breathing difficulties. During a clinical examination, vascular malformation may be suspected if a murmur is detected or a whirl is palpated. USG examination supports the diagnosis if a hypervascularised lesion is demonstrated. Nonetheless, such findings may give the impression of a malignant tumour as part of the differential diagnosis. MDCT, CTAG, potentially MRI or DSA then represent the fundamental methods for determining the diagnosis of TG AVM. Laboratory examination of TG hormones and antibodies is usually within physiological range. Definitive diagnosis from the aspect of malignancy is based on histological examination, which nonetheless is not simple and requires an experienced histopathologist.

TG AVM as part of the Wyburn-Mason syndrome was first described in the form of a unusual case report by the American authors Lee *et al.*[6]. The Wyburn-Mason syndrome represents a hereditary disorder of embryonic ectodermal and neuroectodermal development (so-called phacomatosis), which manifests as the development of vascular malformations of the retina, brain and skin of the face [7, 8]. TG AVM was diagnosed accidentally during angiography and the patient was asymptomatic with normal TG hormone values.

More frequent vascular malformations include haemangiomas, which on USG also give the impression of tumours, presenting as hyperechogenic nodules [9]. In such cases, fine-needle aspiration biopsy (FNAB) is inconclusive, with blood being aspirated in most cases. The definitive diagnostic conclusion can only be made on the basis of surgery and subsequent histopathological examination of the resected tissue. If AVM is suspected, FNAB is not indicated given the important risk of bleeding complications.

## Conclusions

From a pathological point of view, AVM is completely benign and its location within the TG is very rare. Determination of the diagnosis in the case of small lesions is accidental. Clinically more significant manifestations include a mechanical syndrome that places the patient at risk of bleeding complications. Large and symptomatic AVMs require treatment, ideally a combination of radio-interventional embolisation of the arteries supplying the lesion and subsequent surgical resection.

The case report presented here offers an extension of the differential diagnosis options in cases of mechanical neck syndrome, representing a very rare cause mimicking preoperatively a malignant disease. The authors stress the importance of close team cooperation between the endocrinologist, interventional radiologist and surgeon and point out the fundamental aspects of precise preoperative examination and preparation for the surgical procedure itself. The definitive determination of the histopathological diagnosis is not easy and requires a pathologist experienced in the given problem.

### Patient’s perspective

I write the following to provide assistance to the case report written about my operation. I have no medical knowledge or background so I only write from my own perspective and experience.

I began to have nonspecific symptoms as a cough, mild dyspnoea on exertion and non-constant hoarseness a half year ago. I was afraid of having a problem with my heart or lungs, so I decided to visit a cardiologist. The examination showed that my heart is healthy but the cause of my symptoms is deviation of trachea to the right side that was shown on X-ray. I was quickly sent to CT that revealed the possibility of AVM of the TG but did not exclude malignant disease. The same week I underwent the CTAG that gave a more precise finding and showed the main arteries supplying the AVM. The surgeons described the findings to me clearly and recommended the operation, because the findings could be danger by bleeding. They suggested the best way would be cooperation with a radio-invasive procedure which would close the main arteries before surgery and the operation will be for me safer. In 1 month I was received at the hospital, underwent selective intraarterial embolisation and 4 days later I was operated. After both proceuures I felt very well, I was very satisfied and surprised with non-problematic postoperative course. I was discharged home on 7th day after surgery.

The best report I got 2 weeks after operation. It was the result of histological finding that revealed the benign disease. I had no problem with the healing of the wound; nowadays I have a nice scar. These days I go regularly to my endocrinologist, I began to take levothyroxine and my last visit was with ultrasonography of the rest of my TG and is OK.

Now I admire all the specialists who shared in my treatment. They planned the procedures excellently so as to have no complications and my life could be the same as before surgery.

## Consent

Written informed consent was obtained from our patient for the publication of this case report and any accompanying images. A copy of the written consent is available for review by the Editor-in-Chief of this journal.

## Abbreviations

AVM: Arteriovenous malformation
CT: Computed tomography
CTAG: Computed tomography angiography
DSA: Digital subtraction angiography
FNAB: Fine-needle aspiration biopsy
MDCT: Multidetector computed tomography
MRI: Magnetic resonance imaging
TG: Thyroid gland
USG: Ultrasound.
